# Safety, Antagonistic Activity, and Probiotic Properties of Lactic Acid Bacteria Isolated from Jeotgal, Korean Fermented Seafoods

**DOI:** 10.4014/jmb.2411.11055

**Published:** 2024-12-13

**Authors:** Jihyeon Baek, Bong Sun Kim, Yeonju Kim, Jaewoo Bai

**Affiliations:** Department of Food Science and Technology, Seoul Women’s University, Seoul 01797, Republic of Korea

**Keywords:** Jeotgal, *Lactobacillus*, *Leuconostoc*, *Pediococcus*, antibacterial, anti-inflammation

## Abstract

Probiotics are in high demand in the health functional food market as they effectively inhibit pathogens and improve host health. Therefore, in order to develop novel probiotic strains, new strains were isolated from various type of jeotgal, traditional Korean fermented seafood products, and their safety and probiotic properties have been evaluated. Based on 16S rRNA gene sequence analysis, six strains (JRD1, *Pediococcus pentosaceus*; JRD2, *Lactiplantibacillus plantarum*; JRD6, *Pediococcus acidilactici*; CLJ21, *Lactiplantibacillus plantarum*; CLJ24, *Pediococcus pentosaceus*; CLJ28, *Leuconostoc mesenteroides* subsp. *dextranicum*) were selected and subjected to further analysis. As a result, all six strains did not show hemolytic activity, antibiotics resistance, and cell cytotoxicity, confirming that they are safe for human use. Among them, JRD1, JRD6, and CLJ24 exhibited high survival rates under simulated gastrointestinal conditions. Additionally, these three strains demonstrated strong adhesion abilities on HT-29 cells, with values of 6.02, 5.77, and 5.86 log CFU/mL, respectively. Furthermore, JRD1, JRD6, and CLJ24 showed relatively high antagonistic activity against both *Salmonella* Typhimurium and *Staphylococcus aureus* through competition, exclusion, and displacement of their adhesion. Interestingly, cell-free supernatants (CFS) from three strains effectively inhibited the growth of both *S*. Typhimurium and *S. aureus*. Furthermore, CFS of CLJ24, JRD1, and JRD6 demonstrated anti-inflammatory effects in intestinal epithelial cells. The results suggest that CLJ24, JRD1, and JRD6 have potential to be development as functional probiotic strains with both antibacterial and anti-inflammatory activities.

## Introduction

Probiotics are defined by the Food and Agriculture Organization of the United Nations (FAO) and the World Health Organization (WHO) as "Live microorganisms which when administered in adequate amounts confer a health benefit on the host” [1]. Recently, probiotic-based foods have predominated the functional food market, with the global probiotic market expected to expand at an annual rate of approximately 7%, reaching an estimated value of approximately $74 billion in 2024 [2]. Therefore, although several probiotic bacteria are commercially available, meeting increasing consumer demands and providing an ample variety of product choices requires the development of new probiotic strains with health functions [3, 4]. Probiotics mainly include lactic acid bacteria (LAB), *Bifidobacterium*, *Lactobacillus*, *Leuconostoc*, and *Pediococcus*, which are widely known as representative LABs [5]. LABs are generally found in fermented foods such as yogurt, cheese, kimchi, and jeotgal [6]. However, to harness LAB as probiotics, it is critical to establish their safety for host and demonstrate specific properties as probiotics need to be proven [7-9].

Jeotgal is a rich source of microorganisms, especially halotolerant organisms, and is a traditional Korean fermented seafood product primarily made by salting fish, seafood, or their innards [10, 11]. Natural fermentation occurring in jeotgal contributes to the flavor and aroma of jeotgal and promotes the proliferation of various microbial communities [12, 13]. To date, some LAB strains, including *Lactobacillus*, *Leuconostoc*, and *Pediococcus* have been isolated from jeotgal and their probiotic properties have been examined [11, 14-17]. However, researches on their safety, antimicrobial activity, and anti-inflammatory properties are still limited [18-20]. Moreover, probiotic strains isolated from *Changran-jeot* (Alaska pollock intestines) and *jaridom-jeot* (damselfish) have not been studied yet. *Changran-jeot* is a fermented seafood product made by salting and fermenting the intestines of Alaska pollock with seasoning, whereas *jaridom-jeot* is a fermented seafood product made by fermenting damselfish with salt. *Changran-jeot* and *jaridom-jeot* have high salinity levels of approximately 5–8%and 26–31%, respectively [21, 22]. Probiotic strains isolated from foods with high salt content not only have significant salt resistance but also are able to survive under nutrient-poor conditions, allowing them for a variety of applications [14-23]. However, LAB strains isolated from *Changran-jeot* and *jaridom-jeot* have not been reported and studied yet.

LAB strains not only promote intestinal health but also offer health function benefits, including protection against viral infection diseases such as rotavirus and norovirus [24, 25], as well as alleviation of metabolic diseases such as obesity, diabetes, cardiovascular disease, hypertension, and colon cancer [26, 27]. In addition, probiotics exhibit antibacterial activity against pathogenic bacteria by competing for attachment with pathogenic bacteria in the intestinal epithelial region, inhibiting the attachment of pathogenic bacteria to the intestine and producing antibacterial compounds such as organic acids and bacteriocins [28, 29]. Therefore, the antagonistic effects of LAB strains in maintaining and enhancing the overall balance of the intestinal microbiota are important criteria in the selection of probiotics [30]. Recently, research on the functionality of probiotics has gone beyond living bacteria, and research on the health functionality of CFS, such as antibacterial and intestinal immune system regulation by postbiotics (probiotics metabolites) [31].

In this study, we isolated new LAB strains from traditional Korean fermented seafoods, *Changran-jeot* and *jaridom-jeot*, and evaluate their safety. In addition, the antibacterial activity, antagonistic activity of the LAB strains against pathogen, and their anti-inflammatory effects were evaluated. The results showed that new LAB isolates have potential to be used as probiotic strains.

## Materials and Methods

### Isolation of LAB Strains and Growth Conditions

Two traditional Korean fermented seafood samples, *Changran-jeot* (Alaska pollock intestines) and *jaridom-jeot* (damselfish), were collected from the traditional Korean markets: *Changran-jeot* was sourced from the Incheon Sorae Port market, and *jaridom-jeot* was purchased from the Jeju Olle Market. Jeotgal samples (25 g) were mixed with 225 ml of 0.1% peptone water and homogenized for 30 s in a stomacher (LS-400; Bnfkorea Ltd., Republic of Korea). Supernatants of the homogenized samples were 10-fold serially diluted and plated on LAB-selective medium agar. Culture plates were incubated at 30°C or 37°C for 48 h and under aerobic or anaerobic conditions, and colonies were randomly selected. The purity of the isolated strains was confirmed by streaking them twice on fresh de Man Rogosa Sharpe (MRS) medium (BD Difco, USA) and verifying whether the colony was a pure culture. *Lactobacillus rhamnosus* GG (ATCC 53103; LGG) and *L. plantarum* ATCC 8014 (Lp) were used as reference bacterial strains. All isolated strains were stored at -80°C in 25% glycerol (v/v). The isolated bacterial strains were grown for 24 h before use by subculturing them twice in MRS broth.

### Identification of the LAB Strains

LAB strains isolated from jeotgal were identified by performing 16S rRNA sequencing analysis performed by a sequencing service company (Bionics Co., Ltd., Republic of Korea), using the universal primer sets of 27F (5'-AGA GTT TGA TCM TGG CTC AG-3') and 1492R (5'-GGT TAC CTT GTT ACG ACT T-3'). The sequencing results were analyzed using the basic local alignment search tool (BLAST) from the National Center of Biotechnology Information (NCBI, USA) database. The 16S rRNA sequence of each strain was analyzed by blastn with rRNA/ItS databases (bacteria and Archaea) and megablast program.

### Preparation Cell-Free Supernatant (CFS)

First, we cultured three LAB strains in MRS broth at 37°C for 24 h. Following incubation, the cultures were harvested to obtain cell-free supernatant (CFS), which was adjusted to pH 6.5 using 5N NaOH. Subsequently, CFS was subjected to filtration through a 0.45 μm filter. Subsequently, It was confirmed that the CFS formed colonies when streaked on the MRS agar plate. Consequently, the neutralized CFS (pH 6.5) was employed for antibacterial and anti-inflammatory assessments.

### Preparation of HT-29 Cell Culture

Human colon adenocarcinoma cell line, HT-29, was obtained from the Korean Cell Line Bank (Republic of Korea). HT-29 cells are commonly used in probiotics research due to their similarity to human intestinal epithelial cells. HT-29 cells were maintained in Dulbecco’s modified Eagle’s medium (DMEM; Welgene, Republic of Korea) containing 10% fetal bovine serum (FBS; Gibco, Canada), 100 U/ml penicillin, and 100 μg/ml streptomycin (Invitrogen, USA) at 37°C in a 5% CO_2_ atmosphere in a humidified incubator.

### Safety Evaluation of the Isolated Strains

**Hemolysis activity.** LAB strains cultured for 24 h in MRS broth were streaked on Tryptic Soy Agar (TSA; BD Difco, USA) containing 5% (w/v) defibrinated whole sheep blood (Kisan Biotech Co., Ltd., Republic of Korea) and incubated at 37°C for 48 h [32]. The hemolysis activity was observed as partial hydrolysis with grass-green zones around the colonies (α-hemolysis), a clear zone of complete red blood cell lysis around the colonies (β-hemolysis), or no zone around the colonies, indicating no lysis (γ-hemolysis). In addition, *Escherichia coli* O157:H7 ATCC 35150 (α-hemolysis) and *S. aureus* ATCC 27664 (β-hemolysis) were used as positive controls for the analysis of hemolysis [33, 34].

**Antibiotic susceptibility of the LAB strains using E-TEST.** Antibiotic susceptibility of the LAB strains was analyzed using an E-test (Liofilchem, Italy) for the following eight antibiotics, ampicillin, gentamicin, kanamycin, streptomycin, erythromycin, clindamycin, tetracycline, and chloramphenicol according to the European Food Safety Authority (EFSA) guidelines [35]. The concentrations of the antibiotic strips ranged from 0.016–256 μg/ml, with the exception of streptomycin (0.064–1024 μg/ml). Initially, LAB strains cultured at 37°C for 12 h were diluted to approximately 2 × 10^8^ CFU/ml and inoculated on the surface of MRS agar using a sterile cotton swab. After incubation for 20 min under anaerobic conditions, E-test strips were placed on the surface of the inoculated MRS agar plates and incubated anaerobically at 37°C for 48 h. The minimum inhibitory concentration (MIC; μg/ml) was read directly from the scale at the point where the edge of the inhibition ellipse intersected the E-test strips. A microorganism inhibited at the cut-off level for a specific antibiotic was defined as susceptible. If the MIC was higher than the cut-off value, the microorganism was considered resistant. The antibiotic susceptibility of the LAB strains was eventually determined according to the EFSA guidelines. Moreover, the safety evaluation and probiotic characterization of the strains isolated in this study were verified and compared using LGG, a commercially available probiotic strain, as a reference [36, 37]. Additionally, Lp, which is frequently used as a reference strain within the same genus as the LAB strain evaluated in this study, was used as a reference strain [38, 39].

**Evaluation of cytotoxicity.** Cytotoxicity of the LAB strains to intestinal epithelial cells was evaluated using the 3-(4, 5-dimethylthiazol-2-yl)-2, 5-diphenyltetrazolium bromide (MTT) assay (Duchefa Biochemie, Haarlem, Netherlands) [40]. HT-29 cells were added to 96-well plates at 5 × 10^5^ cells/ml and incubated at 37°C for 24 h. After incubation, the cells were treated with each LAB strain resuspended at 1 × 10^6^ CFU/ml in antibiotic-free DMEM containing 10% FBS and incubated at 37°C for 24 h. To induce the reaction, 110 μl of supernatants were replaced with 10 μl MTT reagent (final concentration 0.5 mg/ml) and incubated at 37°C for 4 h in the dark. The culture supernatants were discarded, and the culture plate was gently rinsed with phosphate-buffered saline (PBS). Finally, the formazan crystals were dissolved in 100 μl of dimethyl sulfoxide and the absorbance at 570 nm was measured using a microplate reader (SpectraMax M2e, Molecular Devices LLC, USA).

### Analysis of Probiotic Potential

**Tolerance to simulated upper gastrointestinal transit.** The method of Jensen et al. (2012) was used to assess the tolerance of LAB strains to gastrointestinal transit in simulated gastric and small intestinal juices [41]. Simulated gastric juice was prepared by dissolving pepsin from porcine gastric mucosa (Sigma-Aldrich, USA) in 0.5% w/v NaCl to a final concentration of 2 g/l and adjusting the pH to 3.0. Simulated small intestinal juice was prepared by dissolving 250 mg/l pancreatin from porcine pancreas (Sigma-Aldrich, China) and 0.45% w/v porcine bile extract (Sigma-Aldrich) in 0.5% (w/v) NaCl and adjusting the pH to 7.5. The simulation solutions were freshly prepared on the day of the experiment. The bacterial suspension was mixed with gastric or small intestinal juice and 0.5% (w/v) NaCl, at a final concentration of 1 × 10^9^ CFU/ml. The mixture was then incubated at 37°C for 180 or 240 min with gastric or small intestinal juice, respectively. Following this, the mixture was serially diluted 10-fold to determine the number of viable bacteria. Serial dilutions were plated on MRS agar and incubated at 37°C for 48 h.

**Adhesion of LAB Strains to HT-29 cells.** The adhesion assay described by Kim *et al*. (2019) was used, with some modifications [42]. HT-29 cells were added to 24-well plates at 5 × 10^5^ cells/ml and grown until fully confluent. The LAB strains cultured for 24 h were washed twice with PBS and resuspended in antibiotic-free DMEM containing 10% FBS. Monolayers of HT-29 cells were treated with each LAB strain at a final concentration of 5 × 10^7^ CFU/ml. After incubation at 37°C for 1 h with gentle shaking, HT-29 cells were washed with PBS and lysed with 0.25% Trypsin-EDTA (Thermo Fisher Scientific, USA). The number of adhered LAB strains was determined by plating and counting the cells on the MRS agar.

### Inhibition of Foodborne Pathogenic Bacterial Adhesion to HT-29 Cells

HT-29 cells were plated in 24-well plates at 5 × 10^5^ cells/ml and grown until they were fully confluent. For the competition assay, HT-29 cells were co-treated with each LAB strain (5 × 10^7^ CFU/ml) and the equal number of *Salmonella* Typhimurium ATCC 14028 or *S. aureus* ATCC 6538 in antibiotic-free DMEM containing 10% FBS at 37°C for 1 h with gentle shaking. To test the ability of excluding LAB strains, HT-29 cells were pretreated with the LAB strains (5 × 10^7^ CFU/ml) at 37°C for 1 h. Subsequently, *S*. Typhimurium ATCC 14028 or *S. aureus* ATCC 6538 (5 × 10^7^ CFU/ml) were treated in antibiotics-free DMEM at 37°C for 1 h with gentle shaking. Conversely, for the displacement assay, HT-29 cells were pretreated with *S*. Typhimurium ATCC 14028 or *S. aureus* ATCC 6538 (5 × 10^7^ CFU/ml) at 37°C for 1 h. Then, LAB strains were treated with an equal number in antibiotics free DMEM and incubated at 37°C for 1 h with gentle shaking. After incubation, the HT-29 cells were washed with PBS and lysed with 0.25% trypsin-EDTA (Gibco). The number of adhered bacteria was evaluated by plating cells on selective media. Specifically, Xylose lysine and sodium deoxycholate agar (XLD; BD Difco, USA) were used for *S*. Typhimurium ATCC 14028, and Baird Parker agar (MB Cell, Republic of Korea) supplemented with 5% egg yolk was used to quantify *S. aureus* ATCC 6538.

### Antagonistic Activities to Foodborne Pathogenic Bacteria

The method of Choi *et al*. (2018) was used to determine the antagonistic activity against foodborne pathogens [43]. First, the six LAB strains were cultured in MRS broth at 37°C for 24 h. Following incubation, the cultures were harvested to obtain CFS, which were filtered using a 0.45 μm filter. *S*. Typhimurium ATCC 14028 and *S. aureus* ATCC 6538 were cultured in Luria-Bertani broth (BD Difco) and tryptic soy broth (BD Difco), respectively, for 24 h at 37°C, diluted to 0.06 at 600 nm and 100 μl of each were added to 96-well plates. Further, the CFS of LAB strains (100 μl) was added to each well and incubated for 24 h at 37°C. The effect of CFS on the growth of foodborne pathogenic bacteria was assessed at 600 nm every 3 h.

### Determination of IL-8 Levels by Enzyme-Linked Immunosorbent Assay (ELISA)

HT-29 cells were plated at 4 × 10^5^ cells/ml, and cultured on a 96-well plate until reaching 70–80% confluence [44]. Subsequently, the cells were stimulated with heat-killed *S*. Typhimurium UK1 (1 × 10^7^ CFU/ml) and co-treated with pH 6.5 neutralized 10% CFS (final concentration) derived from the LAB strains for 24 h at 37°C. The secretion of interleukin-8 (IL-8) by HT-29 cells was quantified using a human IL-8 ELISA kit (R&D Systems, USA) following the manufacturer’s instructions.

### Statistical Analysis

All study data are represented as mean ± standard deviation from triplicate experiments. The results of each assay were compared with those of the appropriate control, and the statistical significance was analyzed using an unpaired two-tailed *t*-test using GraphPad Prism 5 (GraphPad Software Inc., USA) or one-way analysis of variance using IBM SPSS Statistics 23 software (IBM, USA).

## Results

### Isolation and Identification of LAB Atrains

Six colonies with different morphologies were isolated and identified using 16S rRNA gene sequencing and the sequence coverage with % identities were analyzed by BLAST (Table 1). The six isolated strains were highly similar to *Lactobacillus plantarum* JCM 1149 (99.9%), *Pediococcus pentosaceus* DSM 20336 (99.7%), *Leuconostoc mesenteroides* subsp. *dextranicum* NCFB 529 (99.9%), *Pediococcus pentosaceus* DSM 20336 (99.8%), *Lactobacillus plantarum* JCM 1149 (100.0%), and *Pediococcus acidilactici* DSM 20284 (99.7%). These LAB strains have been previously isolated from various types of jeotgal [11, 13, 45].

### Safety Evaluation of the Isolated LAB Strains

**Hemolytic activity.** Hemolysis refers to the release of hemoglobin outside the red blood cell membrane due to the rupture and destruction of erythrocyte membranes [46]. According to the FAO/WHO Guidelines for the Evaluation of Probiotics in Food, hemolytic activity is considered an important indicator for the safety of probiotics [1]. Hemolytic activity was categorized into α-, β-, or γ- hemolysis [47, 48]. The six isolated LAB strains did not show a hemolytic circle around their colonies, purporting that they were all γ-hemolytic (Table 2).

**Antibiotic susceptibility of the LAB strains.** The LAB strains used in this study were tested against the antibiotics required by the EFSA using the E-test [49]. All tested LAB strains were resistant to kanamycin and susceptible to ampicillin, erythromycin, clindamycin, tetracycline, and chloramphenicol (Table 3). *L. mesenteroides* CLJ28 was sensitive to all the tested antibiotics except kanamycin (MIC value = 64 μg/ml), which is consistent with previous findings wherein the commercially available starter *L. mesenteroides*-com1 has been found to be sensitive to majority of the tested antibiotics (ampicillin, chloramphenicol, clindamycin, erythromycin, gentamicin, streptomycin, tetracycline, and vancomycin) [50]. Additionally, all strains, except *L. mesenteroides* CLJ28, were resistant to gentamicin, and only the tested *Pediococcus* strains were resistant to streptomycin.

**Fig. 1:**
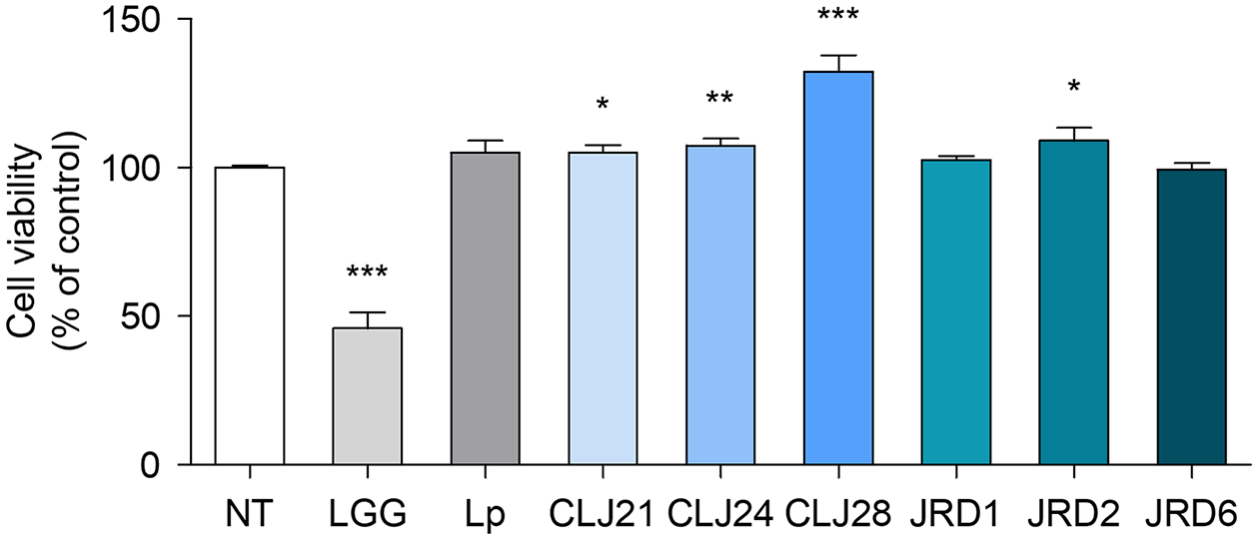
Cell viability of HT-29 cells incubated with the six strains of LAB. JRD1, *Pediococcus pentosaceus*; JRD2, *Lactiplantibacillus plantarum*; JRD6, *Pediococcus acidilactici*; CLJ21, *Lactiplantibacillus plantarum*; CLJ24, *Pediococcus pentosaceus*; CLJ28, *Leuconostoc mesenteroides* subsp. *dextranicum*; LGG, *Lactobacillus rhamnosus* GG; Lp, and *L. plantarum* ATCC 8014. Data are expressed as mean value ± standard deviation of triplicate samples. Asterisks indicate significant differences relative to non-treated cells; *, *p* < 0.05, **, *p* < 0.01, and ***, *p* < 0.001.

**Cytotoxicity of the LAB strains.** The LAB strains were treated on HT-29 cells, to evaluate the effect of LAB isolated from jeotgal on intestinal epithelial cell viability. Cell viability was calculated as a percentage of the LAB strain treatment group relative to that of the control group not treated (NT) with the LAB strains [51]. HT-29 cells treated with the LAB strains showed approximately 100% viability (Fig. 1). None of the tested LAB strains damaged the cells after 24 h of incubation.

### Evaluation of Probiotic Properties

**Tolerance to simulated upper gastrointestinal transit.** The simulated gastric juice tolerance test results revealed high tolerance, with slight growth of *P. pentosaceus* JRD1 and *P. acidilactici* JRD6 (112.99% and 116.74%, respectively) (Table 4). *P. pentosaceus* CLJ24 growth decreased slightly to 97.82% but showed a high survival rate. All LAB strains, except *L. mesenteroides* CLJ28, tolerated the simulated small intestinal juice with pancreatin and bile well with more than 100% viability. *P. pentosaceus* JRD1 and *P. acidilactici* JRD6 showed higher tolerance than those of the reference strain LGG in, both, simulated gastric juice and small intestinal juice. Moreover, *P. pentosaceus* CLJ24 also had a survival rate similar to that of LGG in simulated gastric juice and higher than that of LGG (100.48 %) in simulated small intestinal juice. Thus, *P. pentosaceus* JRD1, *P. acidilactici* JRD6, and *P. pentosaceus* CLJ24 exhibited high tolerance to both gastric and small intestinal juices. *L. mesenteroides* CLJ28 exhibited a 62.84% survival rate following exposure to simulated gastric juice and an 87.91% survival rate following exposure to simulated small intestinal juice. The survival rate was calculated as the percentage of log CFU/ml for the LAB strain treatment group relative to that of the NT group.

**Fig. 2:**
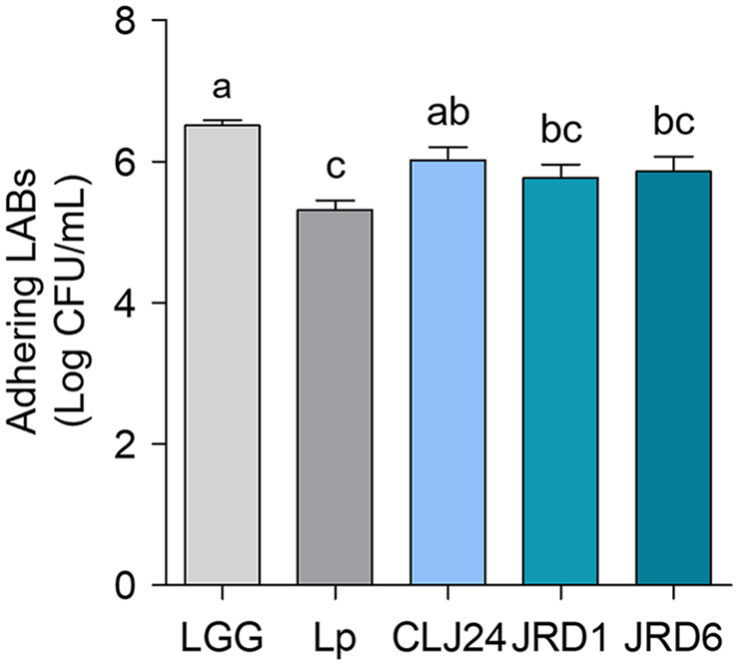
Adhesion of LAB strains isolated from jeotgal to HT-29 cells. Data are expressed as mean value ± standard deviation of triplicate samples. JRD1, *Pediococcus pentosaceus*; JRD6, *Pediococcus acidilactici*; CLJ24, *Pediococcus pentosaceus*; LGG, *Lactobacillus rhamnosus* GG; Lp, and *L. plantarum* ATCC 8014. Values with different superscripts (a–c) are significantly different at *p* < 0.05 using Duncan's multiple range tests.

**Adhesion of LAB strains to HT-29 cells.** The adhesion abilities of CLJ24, JRD1, and JRD6 are shown in Fig. 2. Among the strains tested, CLJ24 (6.02 log CFU/ml) showed the highest adhesive capacity, and JRD1 (5.77 log CFU/ml) showed the lowest adhesive capacity (Fig. 2). Interestingly, the adhesion abilities of the two *Pediococcus* species, CLJ24 (6.02 ± 0.25 log CFU/ml) and JRD6 (5.86 ± 0.30 log CFU/ml) (Fig. 2), were significantly different. This may be because the level of adhesion is strain dependent [52, 53]. The adhesion capacities of the three strains were marginally lower than that of LGG (6.5 ± 0.1 log CFU/ml) and higher than that of Lp (5.3 ± 0.19 log CFU/ml). These results are similar to those of a previous study, in which *P. pentosaceus* and *P. acidilactici* strains showed adhesion levels of 5.21 ± 0.14 and 6.36 ± 0.14 log CFU/ml, respectively [32]. Overall, these findings suggest that, with the exception of JRD2, CLJ21, and CLJ28, which exhibited weak tolerance to simulated gastric or small intestinal juice, all strains showed stronger resistance to simulated upper gastrointestinal transit than LGG or Lp.

### Antagonistic Effect of LABs on Foodborne Pathogenic Bacterial Adhesion in HT-29 Cells

**Fig. 3:**
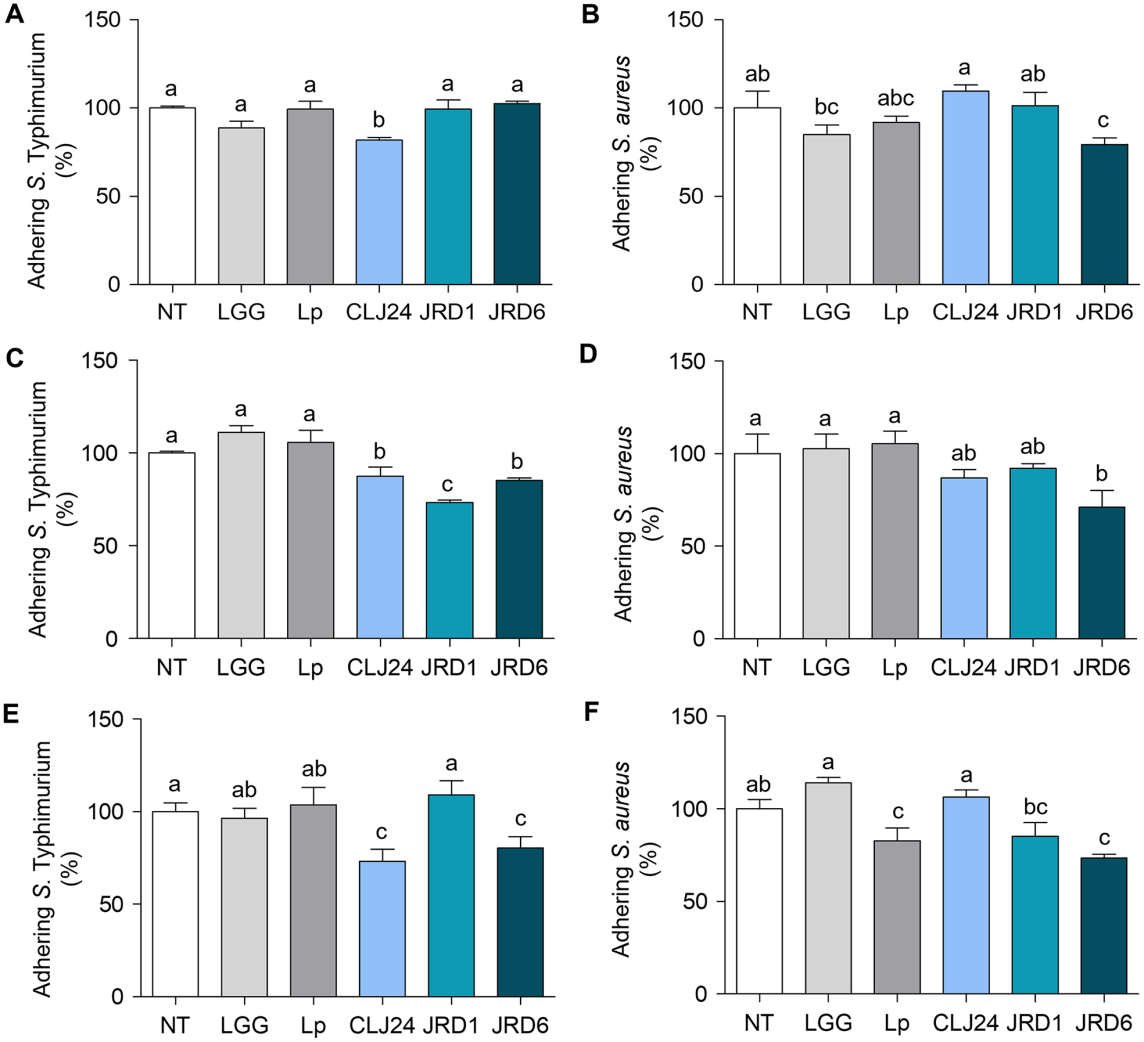
Changes in the adhesion of foodborne pathogenic bacteria to HT-29 cells. HT-29 cells were co-treated with LAB strain and (**A**) Salmonell Typhimurium ATCC 14028 or (**B**) *Staphylococcus aureus* ATCC 6538. (c and d) HT-29 cells were pretreated with each LAB strain followed by treatment with (**C**) *S*. Typhimurium ATCC 14028 or (**D**) *S. aureus* ATCC 6538. (**E** and **F**) HT-29 cells were pretreated with (**E**) *S*. Typhimurium ATCC 14028 or (**F**) *S. aureus* ATCC 6538 followed by treatment with each LAB strain. JRD1, *Pediococcus pentosaceus*; JRD6, *Pediococcus acidilactici*; CLJ24, *Pediococcus pentosaceus*; LGG, *Lactobacillus rhamnosus* GG; Lp, and *L. plantarum* ATCC 8014. Data are expressed as mean value ± standard deviation of triplicate samples. Values with different superscripts (**A-C**) are significantly different at *p* < 0.05 using Duncan's multiple range tests.

In this study, the inhibition of *S*. Typhimurium ATCC 14028 and *S. aureus* ATCC 6538 adhesions to HT-29 cells by the three selected strains (JRD1, JRD6, and CLJ24) was assessed using the following three different methods: competition, exclusion, and displacement. Competition assays demonstrated the ability of the probiotic strains to compete with pathogens for adhesion sites on intestinal epithelial cells when simultaneously treated with pathogens. CLJ24 significantly inhibited *S*. Typhimurium ATCC 14028 adhesion by approximately 18.23%(Fig. 3A). In addition, the adhesion of *S. aureus* ATCC 6538 decreased by 20.55% in the presence of JRD6 (Fig. 3B). These results demonstrate higher inhibitory efficacies than those of the positive controls, LGG, and Lp. Furthermore, exclusion assays showed that once the adherent site of intestinal epithelial cells was occupied by the probiotic bacteria strains, the pathogenic bacteria could not be applied. JRD1 and JRD6 considerably inhibited the adhesion of *S*. Typhimurium ATCC 14028 by 26.7% and *S. aureus* ATCC 6538 by 28.95% when HT-29 cells were pretreated with the LAB strains (Fig. 3C and 3D). In contrast, LGG and Lp increased the attachment of *S*. Typhimurium ATCC 14028 and *S. aureus* ATCC 6538 to HT-29 cells. The displacement assay can remove and displace pathogenic bacteria that initially adhere to intestinal epithelial cells. When HT-29 cells were pretreated with foodborne pathogens followed by the LAB strains, CLJ24 displaced the adhesion of *S*. Typhimurium ATCC 14028 by approximately 26.79% (Fig. 3E). Moreover, JRD6 noticeably displaced *S*. Typhimurium ATCC 14028 by 19.64% and *S. aureus* ATCC 6538 by 26.56%, relative to LGG (Fig. 3E and 3F). This study clearly demonstrated that the JRD1, JRD6, and CLJ24 strains isolated from jeotgal strongly adhered to human intestinal epithelial cells and inhibited foodborne pathogens.

### Antibacterial Effect of CFS from LABs against Foodborne Pathogenic Bacteria

**Fig. 4:**
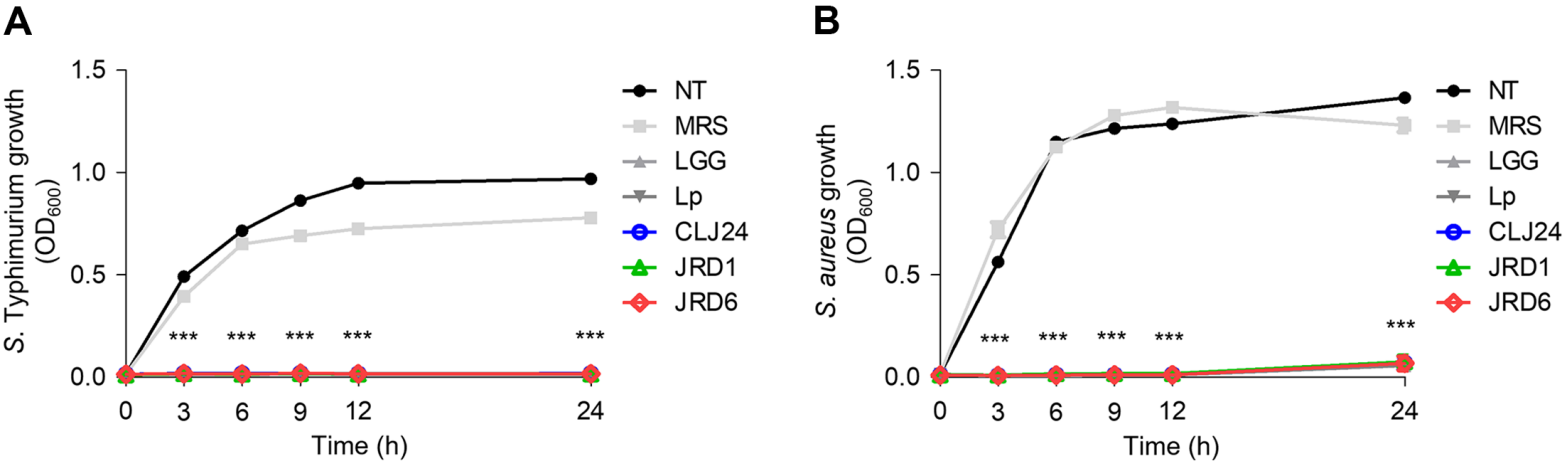
Inhibition of foodborne pathogenic bacterial growth in the presence of cell-free supernatants (CFS) from the LAB strains. (**A**) *S*. Typhimurium ATCC 14028 or (**B**) *S. aureus* ATCC 6538 were incubated in the presence/ absence of CFS from the LAB strains and bacterial growth was determined at OD_600_. ***, *p* < 0.001 compared to NT (nontreatment) with LGG, Lp, JRD1, JRD6, and CLJ24, respectively.

To further investigate the growth inhibitory function of *S*. Typhimurium and *S. aureus* in the presence of the three CFSs, the two bacteria were cultured simultaneously with each CFS. In addition, we compared the MRS medium included in CFS with the vehicle control. As a result, the three CFSs completely inhibited the growth of *S*. Typhimurium and *S. aureus* during the initial stage. Fig. 4A shows that the three CFSs reduced *S*. Typhimurium growth from 3 h of incubation (*p* < 0.05), with a dramatic reduction occurring by 24 h compared to *S*. Typhimurium growth in their absence. Similar to the *S*. Typhimurium inhibition result, the three CFSs showed an initial inhibitory effect on *S. aureus* growth (3 h), after which significant inhibition was detected for 24 h (*p* < 0.05)(Fig. 4B).

### Inhibition on *S*. Typhimurium-Induced IL-8 Production in HT-29

**Fig. 5:**
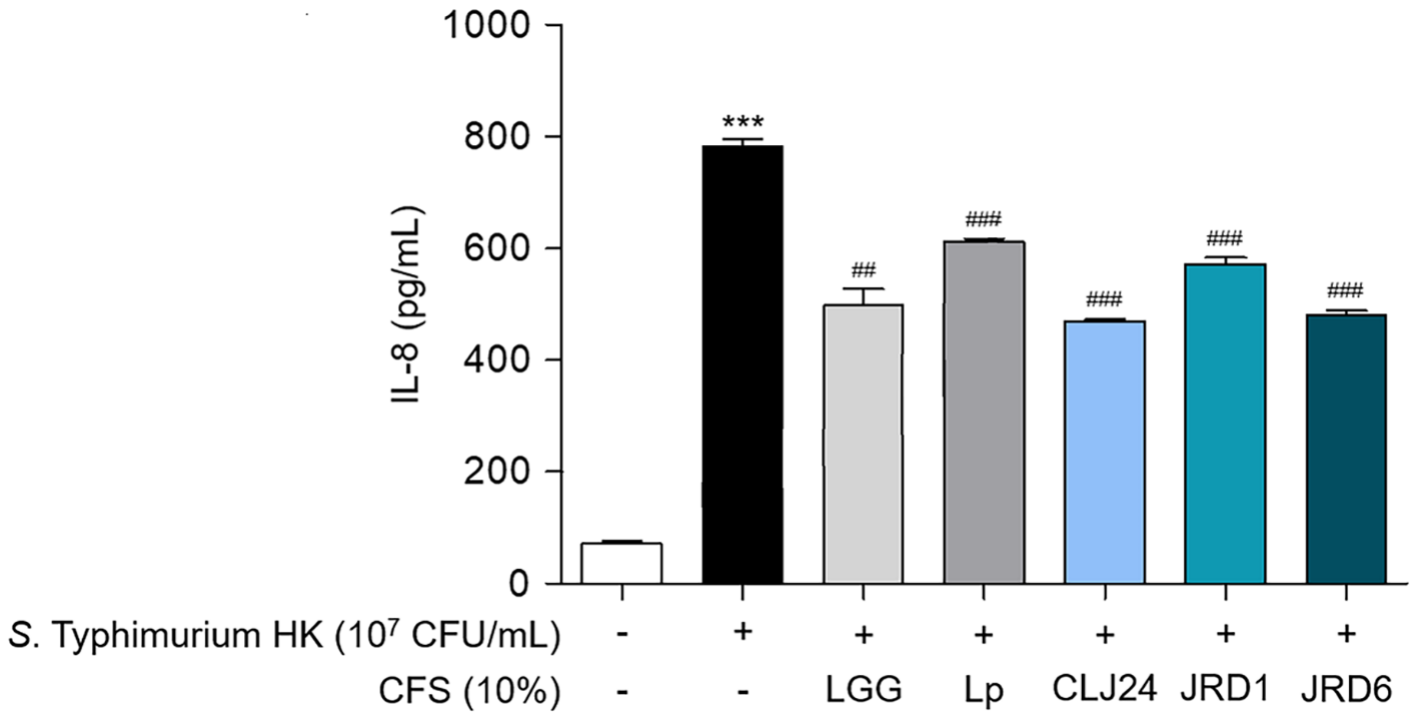
Effects of CFS derived from LAB strains from jeotgal on heat-killed *S*. Typhimurium UK1-induced IL- 8 production in HT-29. HT-29 cells were co-treated with heat killed *S*. Typhimurium UK1 (1 × 10^7^ CFU/ml) and CFS (10%) obtained from LAB strains for 24 h. After treatment, IL-8 secretion was determined using ELISA. Data were expressed as mean value ± standard deviation of triplicate samples. Values with different superscripts (**A**–**D**) are significantly different at *p* < 0.05 using Duncan's multiple range tests.

*S*. Typhimurium induces intestinal inflammation by interacting directly with intestinal epithelial cells, resulting in the secretion of IL-8, a pro-inflammatory cytokine that is critical in recruiting neutrophils and other immune cells to the site of infection or injury [54, 55]. Accordingly, we determined whether CFS from LAB isolated from jeotgal could modulate the increased inflammation caused by *S*. Typhimurium UK1. When heat-killed *S*. Typhimurium UK1 was stimulated, the IL-8 level significantly increased (782.49 ± 24.27 pg/ml) compared to NT (71.64 ± 8.89 pg/ml), confirming an increased inflammatory response (Fig. 5). Moreover, the CFS of three LAB strains (CLJ24, JRD1, and JRD6) down-regulated IL-8 secretion level induced by heat-killed *S*. Typhimurium UK1. The IL-8 levels in three CFS were 469.22 ± 6.99 pg/ml, 572.39 ± 20.25 pg/ml, and 480.43 ± 15.09 pg/ml (respectively, CLJ24, JRD1, and JRD6). This suggests that the CFS of all three LAB strains can effectively improve human intestinal inflammation caused by pathogenic bacteria.

## Discussion

Jeotgal, a traditional Korean fermented food, contains various bacteria. Among these bacteria, probiotics such as *L. plantarum*, *L. mesenteroides*, *P. acidilactici* and *P. pentosaceus* are mainly reported [11, 13, 44]. *L. plantarum* is a versatile LAB found in various habitats, such as the human gastrointestinal tract and different fermented foods [56, 57]. *L. mesenteroides* is the predominant species involved in Korean traditional kimchi fermentation [58]. Bacteria belonging to the genus *Pediococcus*, such as *P. acidilactici* and *P. pentosaceus*, are the predominant species in the LAB community of fermented foods [59]. These LAB species are common probiotic strains widely utilized in the probiotic industry [5, 45, 60-62]. According to existing research, the newly isolated strain proves its potential as a new probiotic strain through probiotic properties analysis, safety verification, and health functional efficacy evaluation [26, 43]. Therefore, in this study, research was conducted on the probiotic properties of *P. pentosaceus* JRD1, *L. plantarum* JRD2, *P. acidilactici* JRD6, *L. plantarum* CLJ21, *P. pentosaceus* CLJ24, and *L. mesenteroides* CLJ28 isolated from traditional Korean fermented foods, *changran-jeot* and *jaridom-jeot*, respectively.

To confirm the safety of the isolated potential probiotics, hemolytic activity, antibiotic susceptibility, and cytotoxicity tests were performed. All safety experiment results of the six isolated strains verified their safety. Firstly, the six probiotic candidate strains isolated did not show hemolytic activity. Additionally, these results were consistent with those of previous studies, wherein *L. plantarum*, *P. pentosaceus*, and *P. acidilactici* strains isolated from *Omegisool* did not show β-hemolysis [32] and no hemolytic activity was detected in *L. mesenteroides* isolated from kimchi [63].

Secondly, the antibiotic resistance of LAB as probiotic strains should be evaluated because antibiotic resistance can be horizontally transferred to intestinal pathogenic bacteria on ingesting antibiotic-resistant probiotic strains [64]. As a result, all LAB strains tested were resistant to kanamycin and sensitive to ampicillin, erythromycin, clindamycin, tetracycline, and chloramphenicol. According to previous research, *L. plantarum* RYPR5, isolated from *Raabadi* and *L. plantarum* S2-6, isolated from Chinese sauerkraut, are resistant to gentamicin and kanamycin, respectively [65, 66]. *P. pentosaceus* SC28, isolated from fermented black persimmon, and *P. acidilactici* OBS 7, isolated from fermented Turkish sausage (*Sucuk*), are resistant to gentamicin, kanamycin, and streptomycin, showing antibiotic resistance types similar to the present study findings [67, 68]. These results indicate a resistance pattern like that of LGG. A previous study on the antibiotic resistance of LGG using the disc diffusion method showed resistance to gentamicin, kanamycin, and streptomycin, which is similar to the antibiotic susceptibility results obtained using the E-test in this study [69, 70]. In addition, Lp showed similar antibiotic resistance to the tested antibiotics, except gentamicin. Most *Lactobacillus* and *Pediococcus* species are intrinsically resistant to aminoglycoside antibiotics, such as gentamicin, kanamycin, and streptomycin. Even though commercially available probiotic strains, such as LGG, also have inherent resistance to antibiotics, this is not thought to pose a significant safety risk as antibiotic resistance genes are unlikely to spread to other harmful bacteria [71-73]. Therefore, it is believed that the newly isolated strain from jeotgal may be safe because it exhibits antibiotic resistance similar to bacteria previously known as probiotics.

Finally, the six probiotic candidates showed non-cytotoxicity to intestinal epithelial cells. Similar results have been reported by Muryany *et al*., (2018) in HT-29 cells treated with *L. plantarum* L8 and *L. plantarum* L20 at 1 × 10^6^ CFU/ml for 24 h [74]. *P. pentosaceus* MZF16 isolated from artisanal Tunisian meat is non-cytotoxic to Caco-2 cells, human intestinal epithelial cells [75]. These findings corroborate the results of this study. LGG exhibited a survival rate of approximately 45.94% in this study, when treated at 1 × 10^6^ CFU/ml. These findings were attributed to the production of extracellular vesicles, including bacteriocins and exopolysaccharides, by LGG, which exert inhibitory effects on cancer cells and induce apoptosis [76]. In summary, the six isolated LAB strains did not exhibit hemolytic activity or cytotoxicity and demonstrated excellent antibiotic susceptibility. Therefore, LAB strains isolated from jeotgal were found to be safe for use as probiotics. Subsequently, further research was conducted to investigate their probiotic properties.

The primary criterion for selecting LAB as a probiotic strain is tolerance to the upper gastrointestinal tract environment. Generally, the stomach forms an effective barrier that prevents the entry of external bacteria at a pH of 2.5–3.5. Food remains in the stomach and is digested under strongly acidic conditions for approximately 3 h [77]. After food passes through the stomach and flows into the small intestine, bile is secreted into the duodenum to aid digestion in the small intestine. The human intestinal tract has a weakly basic environment, with a bile salt concentration of 0.3% (w/v) [78, 79]. Therefore, resistance to extreme conditions such as low pH and bile salts similar to the human intestinal environment is critical for selecting potential probiotic strains [54]. Thus, to determine the probiotic properties of the six newly isolated probiotic candidate strains, we identified their viability in gastric and intestine juice s and their ability to attach their intestines. Among the six candidate LAB strains, *P. pentosaceus* JRD1, *P. acidilactici* JRD6, and *P. pentosaceus* CLJ24 showed high resistance to both gastric and small intestine juice. These results are similar to those reported in earlier studies, wherein the survival rate of *L. mesenteroides* C1 isolated from carrots strongly reduces (approximately 80%) after incubation for 3 h at pH 2.5 and decreases to approximately 90% in 0.3% bile salts [80]. *L. plantarum* GCC_19M1, isolated from fermented milk products, displays a survival rate of 93.48% in simulated gastric juice (pH 3.0) for 2 h, and a survival rate of 83.70% in 0.3% bile for 3 h [81]. In addition, the survival rate of *P. pentosaceus* A24 isolated from Korean jeotgal significantly decreases to 7.5% at pH 3.0 for 2 h, and to 13.7% in 0.3% bile salts [11]. *P. acidilactici* HW01 shows a survival rate of 100.05% in simulated stomach fluid (pH 2.5) over a 3-h period and 97.43% in simulated small intestinal fluid over the same duration [26]. Among the six tested strains, JRD1 and JRD6 exhibited survival rates exceeding 100% in simulated gastric juice, whereas *P. pentosaceus* CLJ24 demonstrated a survival rate of 97.7%, comparable to that of LGG. These results indicated enhanced resistance surpassing the survival rates mentioned earlier for *P. pentosaceus* A24 [11], with a survival rate of 7.5% at pH 3.0 and 13.7% at 0.3% bile salt. Furthermore, these three strains exhibited high viability when exposed to simulated small intestinal juice. Thus, JRD1, JRD6, and CLJ24 can survive sufficiently in the gastrointestinal tract on ingestion, indicating their potential as probiotics. Since all the three strains showed high survival rates in the gastrointestinal tract, which is a critical criterion for probiotic development, further analysis of bacterial binding and adhesion capabilities was performed using these three isolates [82].

JRD1, JRD6, and CLJ24 exhibited adhesion to intestinal epithelial cells, HT-29 cells, as effectively as the positive control. The adhesive capacity of *P. acidilactici* JBNU105117 (8–9 log CFU/ml) isolated from jeotgal on Caco-2 cells is 6.89 CFU/ml, which is lower adhesive than that of LGG (8.17 log CFU/ml), as observed in a previous study [83]. In addition, *P. pentosaceus* L1, isolated from Chinese fermented vegetables (paocai), exhibits significantly lesser capability of adherence to HT-29 cells (6.24 ± 0.46) than that of LGG (12.53 ± 0.58) [59]. Thus, the adhesive capacity of the LAB strains isolated in this study was relatively higher than those of other LAB strains isolated from jeotgal or kimchi. The adhesion ability of LAB is associated with multiple surface components including lipoteichoic acids, polysaccharides, and proteins. In accordance with previous studies, the adhesion ability of *Lactobacillus* strains significantly decreases after trypsin treatment [84]. Consequently, the three strains, JRD1, JRD6, and CLJ24, demonstrating high resistance to simulated gastric and intestinal fluids and optimum adhesion to HT-29 cells, were selected for subsequent experiments. The adhesion is the main characteristic of many bacterial strains used as probiotics [85]. The ability of probiotic bacteria to adhere to the intestine is considered a prerequisite for colonization of the intestine with beneficial bacteria, and the stable colonization of probiotic bacteria in the intestine is the starting point for providing health benefits to the host. The human health benefits of probiotics are primarily known for their antibacterial properties and regulation of intestinal inflammation [77, 86-88]. When infection by pathogenic microorganisms begins in the intestines, pathogenic strains attach to and colonize the host's intestinal mucosal surface, increasing the number of harmful bacteria and leading to intestinal imbalance. At this time, probiotics are known to compete for attachment with pathogens to prevent attachment and colonization of pathogenic strains, and to regulate the secretion of antibacterial active substances and inflammatory mediators through interactions with enterocytes [77, 86, 87, 89, 90]. Using well-established methods, it was confirmed that the adhesive abilities of all three strains tested on HT-29 cells were similar (respectively, CLJ24, JRD1 and JRD6). However, their ability to inhibit the adhesion of pathogenic bacteria varied based on the LAB strain, pathogen, and evaluation method used. This is in agreement with previous studies by Choi *et al*., (2018) and Singh *et al*., (2017), wherein *Lactobacillus reuteri* of fecal origin efficiently inhibited the adhesion of pathogenic bacteria to Caco-2 cells in all three assays, at different levels [42, 87]. Therefore, LAB strains suppress pathogen attachment by preventing colonization through competition, exclusion, and displacement, which are strain-dependent mechanisms in both LAB strains and pathogens [4]. However, the ability of LAB strains isolated from jeotgal to inhibit the attachment of foodborne pathogenic bacteria has not been thoroughly investigated. *L. plantarum* CCMA 0359, isolated from *cauim* can inhibit the adhesion of S. enteritidis to HT-29 cells in both exclusion and competition assays [4]. *L. plantarum* L15 effectively inhibits the attachment of *E. coli* O157:H7 to Caco-2 cells in competition, exclusion, and displacement assays [78]. Pretreatment with JRD1 effectively excluded the adhesion of *S*. Typhimurium ATCC 14028 to HT-29 cells, but pretreatment with *S*. Typhimurium ATCC 14028 did not result in inhibition by JRD1. This suggests that JRD1 does not remove pathogens attached to the surface of intestinal epithelial cells but stably occupies a sufficient number of adhesion sites on the surface of intestinal epithelial cells, thus reducing the adhesion of specific pathogens [91].

Probiotics not only inhibit the adhesion of enteropathogenic bacteria to intestinal cells but also suppress the growth and survival of these bacteria by effectively adhering to the intestinal mucosa. This dual mechanism contributes to the establishment and maintenance of a balanced microbial environment in the intestine [89–90]. The antagonistic activity of probiotics is primarily attributed to the production of antimicrobial compounds such as bacteriocins, organic acids, and hydrogen peroxide [92]. Therefore, this study investigated the effects of CFS produced by LAB strains on the growth of foodborne pathogenic bacteria, specifically *S*. Typhimurium ATCC 14028 (Fig. 4A) and *S. aureus* ATCC 6538 (Fig. 4B). CFS of JRD1, JRD6, and CLJ24 completely inhibited the growth of food poisoning bacteria (*S*. Typhimurium and *S. aureus*) from the early stages of growth. These results are consistent with those of a previous study showing that the CFS from *L. plantarum* MYSAS1 effectively inhibits the growth of *S. aureus* [92]. In general, LAB generate various antimicrobial compounds as well as metabolites derived from fats and amino acids [93, 94]. Based on these findings, although the production of such antimicrobial compounds could not be confirmed, the LAB strains isolated from jeotgal demonstrated inhibitory effects against the growth of foodborne pathogenic bacteria.

Alleviating intestinal inflammatory diseases is a representative functional effect of probiotics [88]. The CFS of certain LAB present in the intestines affects the alleviation of inflammatory bowel disease through various immunomodulatory responses, such as down-regulation of pro-inflammatory gene expression and secretion of chemokines and cytokines in intestinal epithelial cells [95]. For example, CFS of *L. plantarum* T1 demonstrated the ability to hinder lipopolysaccharide-induced cyclooxygenase-2, tumor necrosis factor (TNF), IL-1β, and IL-6 production in RAW264.7 cells [96]. In addition, CFS of *P. acidilactici* TMAB26 effectively inhibited IL-6, IL-10, and TNF-α production in HT-29 cells [97]. On the other hand, previous studies on the regulation of inflammation by strains isolated from jeotgal have focused only on the inflammatory cytokines secreted when the isolated potential probiotics are treated alone [83, 98]. Research like this is not suitable as a model for improving intestinal inflammatory diseases caused by pathogenic bacteria that exist in the intestines. However, unlike previous studies, this result actually confirmed the anti-inflammatory effect of *Salmonella* bacteria that causes intestinal inflammation, proving that it is a probiotic that has the effect of relieving intestinal inflammation. Moreover, in order to scientifically analyze the anti-inflammatory effect of probiotics, the well-known probiotics LGG and Lp were used as comparative standard strains to evaluate the anti-inflammatory effect [44, 99]. Therefore, the level of IL-8 reduction was compared and confirmed using the CFS of LGG and Lp as control strains. Consequently, the IL-8 down-regulation level of CFS of all three strains confirmed to be superior to that of the control strains LGG (498.27 ± 51.88 pg/ml) and Lp (611.94 ± 9.35 pg/ml). As a result, the CFS of all three LAB strains were reduced the expression of IL-8 induced by pathogenic bacteria in HT-29 cells and regulated inflammatory responses more effectively than the CFS of the comparison strains (LGG or Lp), suggesting that these strains are effective probiotics in alleviating enteritis.

## Conclusion

The results of hemolytic activity, antibiotic resistance, and cytotoxicity tests indicated the safety of LAB isolates. Among six isolated strains, JRD1, JRD6, and CLJ24 demonstrated relatively strong resistance under simulated gastrointestinal conditions, surpassing the levels observed in previous studies. Furthermore, these three strains demonstrated strong adhesion abilities and effectively inhibited the attachment of selected pathogenic bacteria to HT-29 cells. Additionally, CFS of three isolated bacteria showed antibacterial activity that inhibited the growth of *S*. Typhimurium ATCC 14028 and *S. aureus* ATCC 6538. Moreover, the three CFSs were observed to have stronger inflammation control efficacy than those of reference strains, LGG and Lp, in HT-29 cells. Although the specific mechanisms for both antibacterial and anti-inflammatory effects of these strains need to be studied, these findings support that the LAB strains isolated in this study have the potential to be used as functional probiotics with high safety.

## Figures and Tables

**Table 1:** Identification of lactic acid bacteria (LAB) isolated from two kinds of jeotgal using 16S ribosomal RNA sequencing.

Isolation source	Strains	BLAST match	GenBank Accession No.	Coverage (% of identity)
*Jalidom-jeot*	JRD1	*Pediococcus pentosaceus* strain DSM 20336	NR_042058.1	100 (99.8%)
	JRD2	*Lactobacillus plantarum* strain JCM 1149	NR_029133.1	100 (100.0%)
	JRD6	*Pediococcus acidilactici* DSM 20284	NR_042057.1	100 (99.7%)
*Changlan-jeot*	CLJ21	*Lactiplantibacillus plantarum* strain JCM 1149	NR_115605.1	100 (99.9%)
	CLJ24	*Pediococcus pentosaceus* strain DSM 20336	NR_042058.1	100 (99.7%)
	CLJ28	*Leuconostoc mesenteroides* subsp. *dextranicum* strain NCFB 529	NR_040817.1	100 (99.9%)

**Table 2:** Hemolysis activity of LAB strains isolated from jeotgal.

LAB	Hemolytic activity
	α/β/γ-hemolysis
*P. pentosaceus* JRD1	γ
*L. plantarum* JRD2	γ
*P. acidilactici* JRD6	γ
*L. plantarum* CLJ21	γ
*P. pentosaceus* CLJ24	γ
*L. mesenteroides* CLJ28	γ
Positive control	
*E. coli* O157:H7 ATCC 35150	α
*S. aureus* ATCC 27664	β

**Table 3:** Minimum inhibitory concentrations (MIC; μg/mL) of antimicrobials for LAB strains determined using the E-TEST.

Strains	AMP		GEN		KAN		STR		ERY		CLI		TET		CHL	
	μg/ml															
	MIC	EFSA cut-off values	MIC	EFSA cut-off values	MIC	EFSA cut-off values	MIC	EFSA cut-off values	MIC	EFSA cut-off values	MIC	EFSA cut-off values	MIC	EFSA cut-off values	MIC	EFSA cut-off values
LGG	0.75	4	128	16	>256	64	96	32	0.094	1	0.19	4	0.38	8	3	4
Lp	0.032	2	4	16	256	64	-^1^	n.r.^2^	0.25	1	0.064	4	3	32	3	8
*P. pentosaceus* JRD1	1.5	4	64	16	>256	64	>1024	64	0.5	1	0.047	1	4	8	4	4
*L. plantarum* JRD2	0.032	2	64	16	>256	64	-	n.r.	0.75	1	1.5	4	4	32	4	8
*P. acidilactici* JRD6	1.5	4	64	16	>256	64	>1024	64	0.5	1	0.064	1	4	8	3	4
*L. plantarum* CLJ 21	0.094	2	64	16	>256	64	-	n.r.	0.38	1	1	4	3	32	4	8
*P. pentosaceus* CLJ 24	2	4	24	16	>256	64	128	64	0.25	1	0.047	1	6	8	4	4
*L. mesenteroides* CLJ28	0.75	2	2	16	64	16	48	64	0.19	1	0.032	1	0.75	8	4	4

Abbreviations of antibiotics are as follows: AMP, ampicillin; GEN, gentamicin; KAN, kanamycin; CD, clindamycin; ERY, erythromycin; TET, tetracycline; and CM, Chloramphenicol. EFSA, European Food Safety Authority; LGG, *L. rhamnosus* GG; Lp, *L. plantarum* ATCC 8014

^1^-, Cut-off value is not established in EFSA guidelines. ^2^n.r., not required

**Table 4:** Tolerance of LAB strains isolated from *jeotgal* to simulated digestive conditions.

	min	Survival rate (%)							
		LGG	Lp	*P. pentosaceus* JRD1	*L. plantarum* JRD2	*P. acidilactici* JRD6	*L. plantarum* CLJ21	*P. pentosaceus* CLJ24	*L. mesenteroides* CLJ28
Gastric juice (pH 3.0)	0	103.44 ± 0.43^a^	107.37 ± 0.50^c^	120.97 ± 0.97^e^	99.13 ± 0.14^f^	114.03 ± 0.64^d^	112.98 ± 0.24^d^	100.73 ± 0.81^g^	104.63 ± 0.0^b^
	180	99.34 ± 0.25^a^	74.78 ± 0.30^f^	112.99 ± 0.24^b^	73.36 ± 0.28^e^	116.74 ± 0.17^c^	92.65 ± 0.32^g^	97.82 ± 0.61^h^	62.84 ± 0.58^d^
Small intestinal juice (pH 7.5)	0	91.47 ± 0.57^a^	106.52 ± 0.33^f^	107.68 ± 0.52^f^	96.94 ± 0.20^b^	101.14 ± 0.30^c^	108.53 ± 0.31^f^	103.85 ± 0.29^d^	103.84 ± 0.23^d^
	240	80.64 ± 0.63^a^	116.90 ± 0.56^fg^	117.65 ± 0.80^g^	103.19 ± 0.00^d^	116.01 ± 0.30^f^	106.44 ± 0.31^e^	100.48 ± 0.29^c^	87.91 ± 0.39^b^

All values are presented as mean ± standard deviation of independent experiments performed in triplicate. LGG, *L. rhamnosus* GG; Lp, *L. plantarum* ATCC 8014

^a-h^ Within the same row, the values for the same simulated juice with different lowercase letters indicate significant differences (*p* < 0.05)
